# Revisiting the B-cell compartment in mouse and humans: more than one B-cell subset exists in the marginal zone and beyond

**DOI:** 10.1186/1471-2172-13-63

**Published:** 2012-11-29

**Authors:** Olivier Garraud, Gwenoline Borhis, Gamal Badr, Séverine Degrelle, Bruno Pozzetto, Fabrice Cognasse, Yolande Richard

**Affiliations:** 1 EA3064–GIMAP, Université de Lyon, Saint-Etienne, France; 2 EFS Auvergne-Loire, Saint-Etienne, France; 3 Vice-Rectorate for Graduate Studies and Research-Visiting Professor Program, King Saud University, Riyadh, Saudi Arabia; 4 INSERM U1016, Institut Cochin, Paris, France; 5 CNRS UMR8104, Paris, France; 6 Université Paris Descartes, Sorbonne Paris Cité, Paris, France; 7 Zoology Department, Faculty of Science, Assiut University, 71516, Assiut, Egypt; 8 Princes Johara Alibrahim Center for Cancer Research, Prostate Cancer Research Chair, College of Medicine, King Saud University, Riyadh, Saudi Arabia; 9 Laboratoire de Microbiologie et Hygiène, CHU de Saint-Etienne, Saint-Etienne, France; 10 Etablissement Français du Sang Auvergne-Loire, 42023, Saint-Etienne cedex 02, France

**Keywords:** BcR, MZ, Breg, B-cells, TLR, Cytokines, Chemokines

## Abstract

The immunological roles of B-cells are being revealed as increasingly complex by functions that are largely beyond their commitment to differentiate into plasma cells and produce antibodies, the key molecular protagonists of innate immunity, and also by their compartmentalisation, a more recently acknowledged property of this immune cell category. For decades, B-cells have been recognised by their expression of an immunoglobulin that serves the function of an antigen receptor, which mediates intracellular signalling assisted by companion molecules. As such, B-cells were considered simple in their functioning compared to the other major type of immune cell, the T-lymphocytes, which comprise conventional T-lymphocyte subsets with seminal roles in homeostasis and pathology, and non-conventional T-lymphocyte subsets for which increasing knowledge is accumulating. Since the discovery that the B-cell family included two distinct categories — the non-conventional, or extrafollicular, B1 cells, that have mainly been characterised in the mouse; and the conventional, or lymph node type, B2 cells — plus the detailed description of the main B-cell regulator, FcγRIIb, and the function of CD40^+^ antigen presenting cells as committed/memory B-cells, progress in B-cell physiology has been slower than in other areas of immunology. Cellular and molecular tools have enabled the revival of innate immunity by allowing almost all aspects of cellular immunology to be re-visited. As such, B-cells were found to express “Pathogen Recognition Receptors” such as TLRs, and use them in concert with B-cell signalling during innate and adaptive immunity. An era of B-cell phenotypic and functional analysis thus began that encompassed the study of B-cell microanatomy principally in the lymph nodes, spleen and mucosae. The novel discovery of the differential localisation of B-cells with distinct phenotypes and functions revealed the compartmentalisation of B-cells. This review thus aims to describe novel findings regarding the B-cell compartments found in the mouse as a model organism, and in human physiology and pathology. It must be emphasised that some differences are noticeable between the mouse and human systems, thus increasing the complexity of B-cell compartmentalisation. Special attention will be given to the (lymph node and spleen) marginal zones, which represent major crossroads for B-cell types and functions and a challenge for understanding better the role of B-cell specificities in innate and adaptive immunology.

## Review and conclusion

### Introduction

A key property of B-cells is that these unique cells of the adaptive immunity branch are the only cells capable of editing epitope-specific tools, i.e., antibodies (Abs), by rapidly superimposing genetic events that end with somatic mutations and class switching to ensure extremely efficient immune responses to a panoply of pathogens, particularly T-dependent (TD) antigens (Ags). Although progress is still being made in understanding the intricate mechanisms of this extremely complex genetic event, this function of B-cells is considered their classical property. Evidence is growing that innate immunity is regaining the importance it probably lost when the much-sought-after function of adaptive immunity and the highly sophisticated role of conventional lymphocytes were finally understood. Intriguingly, while many cell subsets are specific to each branch of immunity, it is now apparent that B-cells have almost equivalent roles in either branch, and their importance as innate immune cells has now been recognised with the discovery of functional and tissue-specific subsets in bone marrow (BM), secondary lymphoid organs and mucosae. B-cells may behave as one-man-bands, using the most appropriate instrument (B-cell receptor, Toll-like receptors, cytokine/chemokine-receptors, etc.) for each situation, but they are also highly plastic in their capacity to produce cytokines. This property was only recently appropriately acknowledged, in comparison to the champion cells for polarising immune responses such as T-cells, dendritic cells (DCs) or macrophages (and now platelets). In hindsight, however, this property could have been deduced from one of the first descriptions of how a lymphocyte was reprogrammed, i.e., after infection with Epstein-Barr virus (EBV) through its unique receptor, CR2/CD21 [1]. Paradoxically, the rediscovery of the roles and functions of B-cell subsets in innate immunity provides the “non-classical” (new) part of the story, which itself is far from being complete, since many discrepancies between mouse and human systems plead for novel extensive research efforts to fill in the gaps.

### Arising questions on B-lymphocytes

Decades ago, lymphocytes were discovered as the major tools of specific immunity, now defined as adaptive immunity, which has unique characteristics based on clonal differentiation and a repertoire of Ag recognition. A long-term paradigm then arose, which postulated that B-cells underwent terminal differentiation into plasma cells and that the majority were then confined to antibody (Ab) production, while some differentiated B-cells were spared from this terminal pathway to constitute a pool of memory B-cells. Memory as well as activated B-cells were then shown to efficiently present Ags to T-cells and undergo mutual activation/differentiation processes that involve cytokines and several pairs of co-stimulatory molecules, including the well-known CD40/CD40L pair [2]. The constitutive expression of CD40 was ascribed first to B-cells, and then extended to most Ag presenting cells (DC and macrophages); this property helped to decipher the Ag presenting cell (APC) function of activated/memory B-cells and their use alongside Major Histocompatibility or MHC molecules [3]. The co-expression of MHC and of CD40 does not, however, licence immune cells as APCs (e.g. platelets possess such characteristics) [4].

B-cells were essentially described for their indispensable role in the so-called humoral (Ab-based) immunity, and the machinery supporting their central functions was thought to be essentially located in the germinal centres (GCs) of lymph nodes and the spleen. Years later, it was acknowledged that this functional microanatomy also concerned the mucosal equivalents of lymph nodes, thus supporting mucosal immunity. With further investigation, however, it became evident that B-cells can exert a number of Ab-independent functions, including capturing and concentrating Ags for presentation, producing cytokines, influencing T-cell and DC responses, contributing towards distinct functions during the immune response *in vivo*, affecting lymphoid tissue structures, and even participating in tissue repair [5].

Around fifteen years ago, the conceptual barrier between specific and non-specific immunity was breached and novel functions were ascribed to innate immunity. As such, the role of almost every immune cell type was revisited. There were few changes to some areas, e.g., conventional T-cells, but novel and important functions have been ascribed to non-conventional T-cells, NK-cells and NKT-cells. The issue of B-cells was much more intriguing for two main reasons: 1) the discovery/identification of non-conventional B-cells; and 2) the identification of non-self infectious danger sensors or receptors (Rcs), which are thought to characterize innate immunity and are abundantly-expressed on B-cells. Indeed, the concept of non-self-infectious danger was subsequently addressed [6], *Pathogen Associated Molecular Patterns* (PAMPs) were described on pathogens, and PAMP-counter ligands, namely *Pathogen Recognition Receptors* (PRRs), were identified on the sensing and sentinel cells of innate immunity. PRRs have remained extremely conserved throughout the evolution of living organisms, with a key family being the Toll-like Rcs or TLRs. We, and others, have further described that a specific pattern of TLRs characterizes every B-cell subset [7-10]. B-cells express Rcs for immunoglobulins (Ig) such as FcγRIIb/CD32 or FcεRII (CD23). CD32 mediates inhibitory signals to BcR-activated B-cells, but also authorizes the internalisation of immune complexes. CD23 is essential for B-cell responses and distinguishes various immature and mature B-cell subsets. Complement Receptor 2 (CR2/CD21) is also found on B-cells, where it amplifies BcR signalling through its association with CD19 and its downstream PI3K-dependent signalling pathway [11]. CD21 also constitutes the unique receptor for EBV, a herpes virus responsible for infectious mononucleosis that is present in more than 85% of healthy individuals in its latent form. Reactivation of EBV is frequently observed in chronically HIV-infected patients where it correlates with a higher frequency of circulating transitional-like B-cells [12]. In addition, EBV^+^ B-cell lymphomas are more frequent in HIV-infected patients than in the general population [13].

The capacity of specific B-cell subsets to traffic throughout the body is essential for sampling pathogens, but also for their APC functions [14]. Accordingly, distinct programs of chemokine receptor expression were also ascribed to the various B-cell subsets [15]. Whereas most B-cells express CXCR4 and CXCR5 throughout the course of B-cell differentiation, their balanced expression is decisive for the emigration of immature B-cells from BM into the spleen where CXCL12, the ligand of CXCR4, is highly expressed in the red pulp [16]. In steady-state conditions, the homing of mature naïve B-cells into follicle anlagen is driven by strong expression of CXCL13, the ligand of CXCR5, within the white pulp [17], whereas the trafficking of memory B-cells beneath CCL20-expressing epithelia is orchestrated by CCR6 [18]. Other chemokine receptors, in association with the highly tissue-specific expression of their ligands, orchestrate B-cell homing into intestinal mucosa (CCR9, CCR10) or the skin (CCR4) in combination with integrins and other adhesion molecules [16,19,20]. BcR or CD40 signals and TLR agonists can tune the expression of chemokine receptors [18,21-23] and therefore impair their trafficking. As such, B-cells that were thought to be almost “rude” cells according to the classical T-cell/B-cell paradigm, are actually split into numerous subpopulations that are functionally and spatially distinct.

Even more recent observations indicate that marginal zone (MZ) and B1-like B-cells are engaged in the innate arm of immune defence [24,25], and that immunoglobulins (Igs) are comprised of two distinct molecular tools: 1) B-cell receptor/”specific” Abs; and 2) instruments of the innate immunity branch, the so-called “polyreactive Igs” that are largely associated with extrafollicular B1-cells [26]. Besides MZ B-cells, recently defined B-cell subsets have also captured the attention, including regulatory B-cells and CD21^lo^ B-cells. Although B-cell phenotype characteristics and physiological functions need to be better defined, recent studies in mice (and also in humans) have clearly revealed their distinguished roles in chronic infection, autoimmune disease and aging. This confirms that B-cells are not only actors but also key regulators in the immune response. This essay thus aims to revisit B-cell immune physiology and reconsider B-cells alongside more recent discoveries.

### Marginal zone B-cells perform a key role in microbial infection

#### The marginal zone: from structure to function

Besides its crucial role in filtering the blood and recycling red blood cell components and iron, the spleen is also the largest secondary lymphoid organ of the body after the discontinuous gut-associated lymphoid tissue (GALT). Whereas the erythrocyte-rich “red pulp” leads to a functionally slow bloodstream that favours blood filtration, the organisation and functions of the lymphocyte-rich “white pulp” closely resemble those found in lymph nodes, with B-cell follicles and T-cell zones [27]. A highly specialised micro-anatomical site surrounding the white pulp, referred to as the MZ, constitutes a unique structure that allows interactions between the key effectors of innate and adaptive immunity.

The MZ is recognised as an anatomical site that enables the cells leaving the bloodstream to transit from the red to the white pulp. The MZ shows prominent structural variation in different animal species: it is well developed in rodents, intermediately developed in primates (human and non-human), and poorly developed in the dog and cat [28]. Whereas the white pulp is specialised for adaptive responses (including Ab-based responses to TD Ags), the MZ is more specialised for the response to blood-borne pathogens (bacteria and viruses), and in particular to T-independent (TI) Ags. This functional distinction between TD and TI responses correlates with the presence of two distinct B-cell populations: follicular and MZ B-cells, respectively. To be fully efficient in inducing an Ab response, the latter B-cells must establish interactions with particular subsets of macrophages, DCs and fibroblast-like cells, and must respond to environmental cytokines in a well-controlled spatiotemporal manner. Mouse macrophages and MZ B-cells, firmly attached to sinus lining cells and reticular fibroblasts bordering the MZ, are kept in close proximity to each other and are locally exposed to blood-borne pathogens. Two discrete subsets of macrophages, the Marginal Metallophilic Macrophages (MMM) and the Marginal Zone Macrophages (MZM), populate the mouse MZ where they perform specialised functions. Both MZM and a subset of MMM express the type I scavenger receptor, MARCO (*macrophage receptor with collagenous structure*) [29]; however, MMM characteristically express sialic acid-binding Ig-like lectin-1 (Siglec-1, Sialoadhesin, CD169) and MZM characteristically express the C-type lectin, SIGN-R1 (*specific intracellular adhesion molecule-3 grabbing non-integrin homolog-related 1, the murine homolog of human DC-SIGN — Dendritic cell-specific ICAM grabbing non integrin/CD209*). MMM are essential for cross-presentation of blood-borne antigens by splenic CD8^+^ DC and initiation of the cytotoxic T-cell response [30], while MZM preferentially act as phagocytic cells responsible for clearing blood-borne pathogens harbouring TI Ags and apoptotic material entering the spleen [31,32]. Once MZM bind pathogens, they establish the direct cell–cell interactions with MZ B-cells required for an efficient Ab response [33,34]. As shown in various models of deficient mice, B-cells are reciprocally required for normal MZ development and maintenance as well as for the presence and functions of MZM [29]. Even in the transient absence of MZ B-cells following administration of LPS or FTY720 (a drug that blocks emigration from lymphoid organs into blood and lymph), MZM quickly lose SIGN-R1 expression and phagocytic activity [35]. In turn, the binding of pathogens to MZ B-cells and the IgM response to polysaccharides only occur when SIGN-R1-expressing MZM are present to first capture pathogens [36]. Moreover, loss of MZM and MMM (by treatment with clodronate-containing liposomes) leads to MZ B-cell loss and impaired trapping of particulate Ag. After treatment, the recovery of MZ B-cells is delayed until MZM and MMM fully repopulate the MZ [35]. In the absence of MZ B-cells, CD11c^+^ dendritic cells normally concentrate in the MZ bridging channels and are redistributed around the MZ, which likely impairs the adaptive immune response as a consequence [37]. Thus, a constant dialogue between MZ B-cells and MZM, and likely with MMM, is mandatory for protecting intact MZ architecture, as well as for efficient clearance of pathogens and immune responses. MZM and MMM also constitute important sources of type I interferon (IFN) during murine infection by HSV (*Herpes Simplex Virus*) and LCMV (*Lymphocytic Choriomeningitis Virus*), which control virus replication and induce anti-viral responses [38,39]. This is consistent with type I IFN promoting functional activation of DC and cross-priming of CD8^+^ T-cells in LCMV-infected mice [40,41].

**Figure 1:**
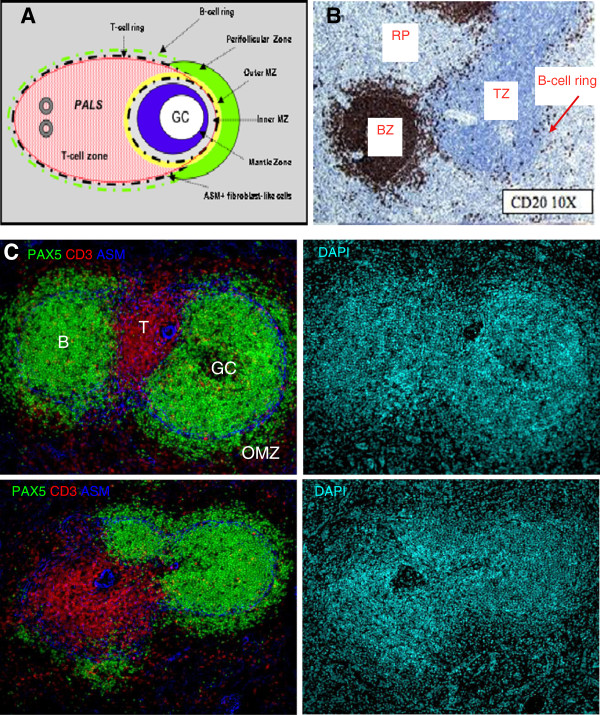
**Organisation of the follicular and MZ B-cell compartments in the human spleen.** (**A**) Schematic representation of the various T- and B-cell areas in the human spleen. PALS: periarteriolar lymphatic sheath (T-cell zone). (**B**) Staining of paraffin-embedded sections of human spleen with CD20 mAb revealed B-cell follicles (BZ) and a ring of B-cells separating the T-cell zone (TZ) from the red pulp (RP) *(original magnification x10)*. (**C**) Sections of human spleen were simultaneously stained with PAX5, CD3 and ASM (alpha smooth muscle actin) mAbs. The network of fibroblast-like cells stained with the anti-ASM mAb (blue) subdivides the outer (OMZ) from the inner marginal zone around B-cell follicles (PAX5+, green) and separates the T-cell zone (CD3+, red) from the RP (left panels) *(original magnification x10)*. In the upper left panel, a germinal centre (GC) is visible within the B-cell follicle. General tissue organisation is shown by DAPI staining of nuclei (right panels).

In contrast to the mouse, the human spleen lacks a marginal sinus, and MZ surrounds B-cell follicles but not the PALS (*Periarteriolar Lymphatic Sheath* or T-cell rich area). However, the perifollicular zone is an intermediate area between the MZ and the red pulp (Figure 1). This zone presents strong similarities to the red pulp because of terminal sinuses, blood-filled spaces, sheathed capillaries without endothelial lining, and scattered B- and T-cells. Because terminal vessels directly open into the perifollicular zone, Ags and leukocytes likely exit the circulation in this structure and traffic either to the MZ or to the red pulp [42,43]. A meshwork of fibroblast-like cells expressing *Alpha Smooth Muscle* actin (ASM) and MadCAM-1 (*Mucosal addressing cell adhesion molecule 1*) subdivides the MZ into a large inner and a small outer compartment, the latter being in close contact with the perifollicular zone. Along the network of ASM-positive cells, a small ring of B-cells delimits the T-cell zone from the red pulp, whereas a ring of T-cells is frequently present between the inner and outer MZ [44]. The MZM and MMM subsets that respectively populate the outer and inner MZ in mice are lacking in humans. However, human macrophages expressing CD68 (lysosome/macrosialin) and CD169 preferentially form sheaths around capillaries in the perifollicular area, and they can also be present as scattered cells expressing DC-SIGN [45,46]. Whether these macrophages may replace MZM in trapping pathogens and interacting with MZ B-cells remains to be established. In humans, CD11c^+^ CD205^+^ DCs are also intertwined with MadCAM1^+^ cells at the inner border of the perifollicular zone, whereas BDCA-2^+^ plasmacytoid DCs are present in the MZ and T-cell zones under steady-state conditions [47-50].

#### Marginal zone B-cells are essential for MZ organisation and functions

When mature B-cells are gradually depleted experimentally in mice, or when BcR signalling is impaired from the time of birth, MMM and SIGN-R1^+^ MZM are absent from the MZ, leading to the decreased expression of MadCAM-1 in the marginal sinus and to a lack of response to TI Ags [29,35,51]. MadCAM-1 expression, which is essential for proper trafficking of DC and macrophages in the MZ during immune responses and normal marginal sinus organisation, depends on permanent interactions between membrane lymphotoxin β (LTβ) expressed by lymphoid cells and its receptor expressed by sinus lining endothelial cells [52,53]. While MZ B-cells can provide LTβ signals that induce MadCAM-1 expression during adult life, naïve follicular B-cells and as yet unknown non-lymphoid cells deliver signals requested during mouse and likely human neonatal life [53]. Recent data on subcutaneous infection of mice by VSV (*Vesicular Stomatitis Virus*) have demonstrated that CD169^+^ macrophages in the subcapsular sinus of lymph nodes have neuroprotective functions [54]. Similar to the situation in the spleen MZ, B-cell derived LTβ renders these CD169^+^ macrophages capable of replicating VSV and of producing type I IFN. Therefore, B-cells exert a critical innate function in antiviral immunity by maintaining the specific location, phenotype and functions of CD169^+^ macrophages in the spleen MZ as well as in the subcapsular lymph node sinus according to the route of virus entry.

In addition to LFA1 and α4β1 (VLA-4, *very late antigen-4*) integrins [55], S1P1 and S1P, receptors 1 and 3 of sphingosine-1-phosphate, contribute to the retention of MZ B-cells in the MZ, whereas follicular B-cells expressing higher levels of CXCR5 freely circulate towards the white pulp [56]. Thus, the balance between sphingosine-1-phosphate and CCL21/CXCL13 plays a determining role in the integrity of the marginal sinus and the retention of MZM and MZ B-cells [57-59]. Impaired CCL21 production correlates with a loss of MZM and reduced pathogen clearance after infection by *Leishmania donovani*[57,60]. These data clearly demonstrate that the proper positioning of MZ B-cells is strongly dependent on the integrity of the sinus-lining cells [52], and that B-cells are crucial for the development (follicular B-cells) and maintenance (MZ B-cells) of a functional spleen MZ or its equivalent in the subcapsular sinus of lymph nodes.

#### MZ B-cells: a particular subset of memory B-cells in humans

**Figure 2:**
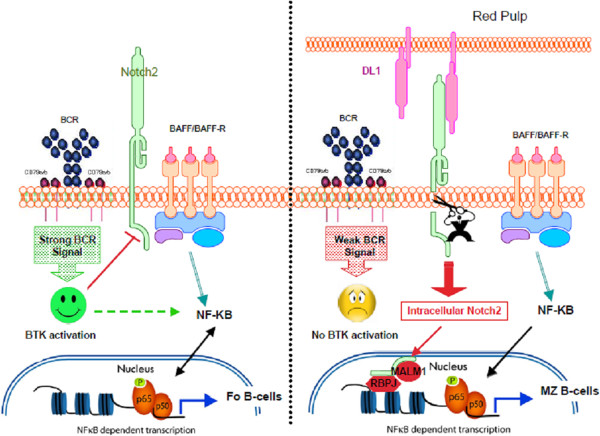
**Becoming a follicular or a MZ B-cell?. Left panel:** Strong signalling through BcR activates Bruton's tyrosine kinase (BTK), which in turn activates the canonical nuclear factor-kB (NF-kB) signalling pathway and prevents the cleavage of Notch2. BAFF-BAFF-R interactions deliver survival signals through NF-KB activation. **Right panel:** Notch2 can interact with its ligand, Delta-Like 1 (DL1), specifically expressed by the endothelial cells of red pulp venules in mice. This interaction initiates the cleavage of Notch2, which is not inhibited by weak BcR signalling. The intracellular domain of Notch2 enters into the nucleus where it interacts with Mastermind-like 1 (MAML1) and RBP-J transcription factors. This transcriptional complex induces the commitment of B-cells towards MZ B-cells. BAFF-BAFF-R interactions deliver survival signals through canonical NF-KB activation.

In mice, orientation towards follicular or MZ B-cell differentiation pathways likely depends on the interplay between BcR and Notch2 signalling. Although absent from the BM, Delta-like 1, a ligand of Notch2, is expressed by splenic red pulp venules and possibly by some stromal cells. No MZ B-cells develop in mice conditionally deficient for Notch2 or Delta-like 1 [61,62]. Follicular B-cells develop in a Notch-independent, but a BcR- and BtK-dependent, fashion [63,64]. As shown by Loder et al., the progression from immature B-cells into mature B2 B-cells is accompanied by a proliferative burst and is governed by a BcR-mediated selection process [65]. According to the “signal-strength” model proposed by Pillai and co-workers, the strength and duration of the BcR signal play a determining role in the differentiation of either follicular or MZ B-cells (Figure 2). Indeed, when self-antigens trigger BcR poorly, the downstream BtK signalling pathway is insufficient to prevent transcription of Notch2-related genes. BAFF (*B-cell activating factor of the TNF family*) then allows the survival of these B-cells, which thus differentiate into MZ B-cells. Conversely, when BcR is strongly triggered by “high-affinity” self-antigens, B-cells differentiate in a BtK-dependent manner into follicular B-cells [61,63,66]. Reciprocally, only strong BcR signals leading to potent Btk activation can counteract the Notch2-induced cleavage initiated by Delta-like 1 (Figure 2). Therefore, weak signals delivered by the BcR appear to be permissive for MZ B-cell development [61,66].

Mouse and human MZ B-cells can be distinguished from follicular naïve B-cells as being SIgM^hi^CD21^hi^SIgD^lo^ and CD23^-^. In humans, MZ B-cells are CD27^+^ memory B-cells that express somatically mutated Ig V_H_ genes [67-69]. As compared with naïve B-cells, blood and spleen MZ B-cells harbour shorter length V_H_ CDR3 in adults but also in young infants, indicating that selection can take place in the absence of antigenic responses [70]. By means of H-CDR3 spectra typing on B-cell subsets present in CD40- and AID-deficient infants compared with age-matched healthy infants, Weller et al*.* have established that MZ B-cells mutate their BcR independently of Ag driven immune responses [69,71,72]. The frequency of V_H_ gene mutation in blood MZ B-cells reaches adult values by two to four years of age, and MZ B-cells express a higher clonal diversity than in other memory B-cells in adult individuals [69,71]. These authors therefore suggested that MZ B-cells express a “pre-diversified” Ig repertoire specialised in their response to TI Ags [69,72]. BAFF (produced in the MZ) has been proposed to be a key contributor to the diversification of the MZ B-cell repertoire through Activation Induced cytidine Deaminase (AID)-dependent mechanisms [73]. However, only very rare MZ B-cells express AID in human spleen: this suggests that alternative processes exist (although they have not yet been identified) [74]. BAFF is a key survival factor for MZ B-cells that also favours the survival of self-reactive B-cells when it is overproduced locally in response to viral infection or chronic inflammation [75]. Deregulated BAFF expression is a feature of numerous autoimmune diseases such as rheumatoid arthritis, lupus erythematosis and Sjögren’s disease, where functional BAFF would promote the emergence/survival of self-reactive pathogenic B-cells [75]. In patients with Sjögren’s disease, where BAFF is locally produced by salivary epithelial cells and infiltrating CD8^+^ T-cells [76], BAFF levels correlate with autoantibody levels in serum [77]. Mice transgenic for BAFF can develop Sjögren-like syndromes characterised by expansion of pathogenic B-cells with an MZ-like phenotype. Depletion of MZ B-cells totally prevents this syndrome but does not prevent nephritis, which is also observed in these mice [78]. Consistent with a possible association between viral infection and Sjögren syndrome, Ittah *et al.* have shown that triggering of TLR3 by synthetic agonists or dsRNA does induce exaggerated BAFF production by salivary gland epithelial cells [79]. More recently, these authors have suggested a comprehensive role of viral infection in BAFF production by epithelial cells and monocytes through Type I IFN-dependent and independent mechanisms [80]. In macaques experimentally infected with a pathogenic strain of Simian Immunodeficiency Virus (SIV), we have recently described elevated plasma levels of BAFF, but not APRIL (*A Proliferation-Inducing Ligand*), during the acute phase of infection [81]. The proportions of MZ B-cells strongly decreased in blood, lymph nodes and spleen because of increased apoptosis and their differentiation into polyclonal plasma cells located in the MZ and lymph node sinus, where BAFF over-expression was concurrently observed [81,82]. Therefore, cognate interactions with the virus and virus-induced cytokines likely synergize to promote MZ B-cell differentiation into multi-reactive plasma cells during acute SIV infection. In contrast, the over-expression of BAFF within GCs would impair the selection process of SIV-specific plasma cell precursors, contributing to the delayed SIV-specific antibody detection [81,82]. We are currently addressing this question in SIV-infected macaques treated or not with BR3-Fc, a BAFF antagonist currently used for treating patients with rheumatoid arthritis or lupus erythematosis.

#### Marginal zone B-cells: a dual role in T-independent and T-dependent responses

**Figure 3:**
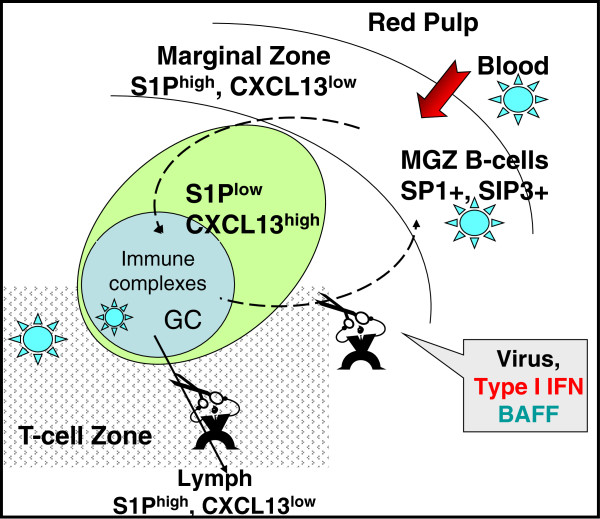
**MZ B-cells shuttle between the MZ and follicles and transport Ag and pathogens to follicular DC.** In steady-state conditions, strong expression of LFA1 and α4β1 integrins and receptors 1 and 3 of Sphingosine 1-Phosphate (S1P) on MZ B-cells, together with high levels of S1P in blood, contributes to the retention of MZ B-cells within the MZ. Type I IFN produced in response to blood-borne pathogens inactivates S1P1 and 3, allowing MZ B-cells to migrate in response to CXCL13, which is highly expressed in follicles. During this relocation, MZ B-cells can transport immune complexes bound to non-BcR receptors and deliver them to follicular DC (FDC). Once on FDC, these import Ags participate in the adaptive Ab response [56,83]. Rapid ligand-induced desensitisation of CXCR5 authorizes MZ B-cells to return to the MZ. Overproduction of BAFF, which preferentially increases the chemotaxis of CD27+ MZ and memory B-cells to CXCL13 might also impair this shuttling and lead to prolonged sequestration of MZ B-cells within follicles [86]. Such a mechanism would be at work during acute infection by SIV, where it might favour sequestration of activated B-cells within follicles [82].

Interactions of MZ B-cells with pathogens (particularly bacteria) elicit their rapid activation and secretion of IgM, which constitutes the first line of defence against these pathogens. The IgM molecules produced are of low affinity but exhibit a broad specificity, meaning that they can bind a variety of Ags with overlapping or close epitopes, favouring the neutralisation and clearance of a larger variety of (bacterial and viral) pathogens. In addition to their role in the innate response, MZ B-cells are endowed with the ability to import high molecular weight Ags (>200 kDa) and viral particles into follicles, a mechanism that can accelerate the adaptive response against pathogens and widen the repertoire of Ags present in GC [56]. In most cases, stimulation of these MZ B-cells occurs independently of the BcR, through triggering of TLRs or other PRRs. Through their capacity to shuttle between MZ and B-cell follicles, MZ B-cells rapidly transport IgM-containing immune complexes delivering pathogen-derived Ags and/or viruses into B-cell follicles where they can rapidly stimulate the onset of primary TD responses [56,83]. Under physiological conditions, MZ B-cells re-enter the MZ once they have delivered Ags to follicular DC within GCs [56,83]. During chronic infection or inflammation, over-production of type I IFN plays a major role in the long-lasting sequestration of B-cells within the follicles by modifying their responsiveness to S1P [84,85]. This B-cell sequestration would favour Ag delivery to follicular DC and interactions between rare T- and B-cells with identical Ag specificity at the border of follicles [56]. Conversely, long-term exposure to type I IFN might have immunosuppressive effects on the B-cell response by interrupting the shuttling of MZ B-cells into the white pulp. Besides type I IFN, we have shown that BAFF might prolong the follicular sequestration of MZ B-cells through its ability to preferentially enhance the chemotaxis of CD27^+^ memory B-cells to CXCL13, [86]. This might particularly occur during HIV/SIV infection [82] or chronic inflammatory diseases where type I IFN and BAFF are concurrently over-produced (Figure 3).

In summary, MZ B-cells, which primarily mount TI responses with so-called “natural” IgM Abs of low affinity and large specificity to a variety of pathogens, can also migrate to follicles where they contribute, via harboured IgM/pathogen complexes, to the initiation of another type of Ab response (that of high affinity/restricted specificity) requiring cognate interactions with helper T-cells.

#### Evidence for a protective role of marginal zone B-cells in systemic bacterial infection

##### MZ and microbial responses in infants

Consistent with data in mice showing that the marginal sinus undergoes organisation during the three first weeks of life [53], ASM expression in infants is rapidly observed around B-cell aggregates after birth. This “rudimentary” MZ organisation attains the adult morphology only after 4 months [87]. Based on the expression of CD21 or CD1c, conflicting data have been reported regarding the colonisation of MZ by MZ B-cells before 2 years of age. Indeed, the group of W. Timens reported that naïve but not CD21^+^CD27^+^ B-cells were present in the MZ prior to the age of 2 years, and they additionally showed that MZ B-cells increased in numbers between the ages of 2 and 5 years, where they reached adult levels [87]. Moreover, even if switched CD27^+^ memory B-cells can be generated in vaccinated infants at 4 to 6 months after birth, they are unable to home into MZ as they do in adults. Responses to polysaccharides occur concurrently with the expression of CD21 in the MZ [88]. Therefore, cellular components other than MZ B-cells are probably absent or not fully functional before the age of two, which limits their homing/sequestration in MZ. In contrast, Weill *et al.* observed CD1c expression in the spleen MZ in 8-month-old infants; they also consistently detected B-cells with a MZ B-cell phenotype in spleens of infants below 2 years of age [72]. However CD1c was previously demonstrated to be expressed by about 50% of spleen and blood B-cells, and to be consistently present on mantle zone, likely naïve, B-cells [89]. While definitive conclusions regarding the kinetics of MZ colonisation by MZ B-cells require additional studies, it is clear that MZ is not fully functional below 2 to 4 years of age, an observation consistent with high susceptibility of children less than 2–3 years of age to infections by encapsulated bacteria and defects in long-lasting protection towards the polysaccharide pneumococcal vaccine [90,91]. Bearing in mind the importance of the dialogue between MZ B-cells and either macrophages or DC subsets in mice, one can speculate that the homing of these populations within the human MZ plays a similar crucial role in MZ function. However, little is known about the kinetics and the precise nature of DC and macrophages that populate the MZ during the first months of life and their capacity to express integrins, BAFF/APRIL, and chemokines known to regulate the homing and survival of MZ B-cells *in situ*. Neutrophils may play a previously underappreciated role as potent producers of BAFF/APRIL and IL21 in the perifollicular zone of human spleen that supports Ig class switching, somatic mutation and Ab production by MZ B-cells [92].

##### Lessons from aging, splenectomy and diseases

Aging in humans and mice is associated with a decline in protective immunity and increased susceptibility to infection by encapsulated bacteria, increased autoimmune manifestation, and reduced response to vaccination. Decreased responses to TI antigens in old mice have been associated with reduced proportions of MZMs and MZ B-cells, collapse of MZ architecture, and disturbed MadCAM-1 expression by sinus lining cells [52]. In humans, invasive pneumococcal disease increases with age, over 65 in particular [93]. The inefficient Ab response in aged individuals is thus assumed to be associated with altered functions of the spleen MZ [88,94]. Individuals with splenic dysfunction or having undergone splenectomy are highly susceptible to infection with *Streptococcus pneumonia, Heamophilus influenza*, *Neisseria meningitis* and other encapsulated bacteria, which correlates with a defect in circulating MZ B-cells and a lack of Ab response against these pathogens [95]. In patients with common variable immunodeficiency, those presenting with recurrent infection of the respiratory tract and chronic lung disease have an extremely low frequency of IgM memory B-cells and do not produce anti-polysaccharide IgM [96,97]. Splenic dysfunction may also occur in patients with sickle cell anaemia [98], inflammatory bowel disease [99] or celiac disease [100], where both impaired IgM production by MZ B-cells and reduced phagocytosis of opsonised particles prevent the clearance of encapsulated bacteria.

Taken together, these observations indicate that the spleen exerts a dedicated function that is not completely substituted by other secondary lymphoid organs. It is noteworthy, however, that a normal blood MZ B-cell compartment is present in young children with congenital asplenia, suggesting that MZ B-cell precursors can colonize alternative sites for their development [69]. Similarly, cells with the morphology and phenotypes of MZ B-cells have been observed in the subcapsular sinus of lymph nodes, under the dome epithelium of Peyer’s patches in the gut, and in the crypt epithelium of tonsils [101-104]. Whether these cells have a similar role to spleen MZ in the defence against encapsulated bacteria remains to be established. Recent data showing the unique ability of B-cells to maintain the location and functions of subcapsular CD169^+^ macrophages during innate antiviral immunity strongly support this hypothesis [54].

### Human B-cells express Toll-like receptors that define anatomical and functional subsets

TLRs are commonly divided into two subgroups depending on their cellular localisation and respective PAMP ligands. The first group is composed of TLR1, TLR2, TLR4, TLR5, TLR6 and TLRs 10–13, which are expressed on cell surfaces and recognize mainly microbial surface components. The second group is composed of TLR3, TLR7, TLR8 and TLR9, which are preferentially sequestered in the endoplasmic reticulum in resting cells and rapidly traffic to endolysosomes after stimulation by microbial nucleic acids [105]. In contrast to mouse B-cells, human B-cells express neither TLR4 nor CD14, the two canonical ligands for Gram^–^ bacteria LPS, and are therefore unresponsive to LPS. In healthy donors, circulating naïve and memory B-cells express distinct amounts of TLR1, 2, 6, 7, 9 and 10 and are negative for TLR 3–5 and 8 [3,10,106]. However, elegant studies by Cerutti’s group have clearly shown that functional TLR3 is expressed by human tonsillar B-cells, with higher expression in GCs and sub-epithelial regions, but is absent from memory B-cells. In the presence of dsRNA, these mucosal B-cells up-regulate AID expression and initiate class switch recombination and IgG/IgA production in the presence of IL-10 and BAFF [107]. Restricted to a discrete population of CD27^–^ blood B-cells with intermediate levels of CD19, TLR2 is preferentially expressed by naïve and GC B-cells present in mucosa or exposed to inflammatory stimuli. MZ B-cells, as well as mucosal follicular naïve B-cells, strongly express TLR2/TLR1 and TLR2/TLR6 complexes and thus recognize a panoply of unrelated molecules, including peptidoglycans, diacylated and triacylated lipopeptides, and porins from a broad spectrum of microbes [108]. BcR cross-linking with anti-Ig antibodies or protein A from *Staphylococcus aureus* sensitizes B-cells to TLR2-active diacylated and triacylated lipopeptides, which promote their proliferation and differentiation into IgM-producing cells [106,109]. Accordingly, TLR2 ligands are effective adjuvants in humans and are currently used in the *Haemophilus influenzae* type B vaccine. *In vitro*, TLR2 ligands also induce CCR9, CCR10 and J chain expression on human B-cells and enhance IgA production [23], suggesting that TLR2 activation might favour intestinal homing. It is not clear yet whether this effect ameliorates the mucosal response or induces tolerance.

In humans, TLR9 expression is restricted to B-cells [10], plasmacytoid DCs (pDCs) [7] and platelets [110], whereas TLR7 is more widely distributed among the various populations of APCs. TLR7 and TLR9 are consistently present in all B-cell subsets, with the majority of TLR molecules sequestered in the endoplasmic reticulum. Activation of TLR9 requires its cleavage by endolysosomal proteases [111], and TLR9 is detectable within small intracellular vesicles of primary B-cells [112]. Memory B-cells contain higher amounts of TLR9 than naïve B-cells [113], and we found that a fraction of TLR9 molecules can traffic to the plasma membrane [10]. BcR- and CD40-mediated stimulation transiently increases TLR9 expression, which enhances responsiveness to its agonist, CpG DNA, proliferation, chemokine production and APC function [7,114]. Besides BcR and CD40 ligation, Type I IFN produced by pDCs increases TLR7 and MyD88 expression in naïve peripheral human B-cells [115], and therefore their responsiveness to TLR7 agonists. Mouse TLR10 is not functional because of a retroviral insertion, and TLR11–13 have been lost from the human genome [105]. While TLR10 expression is also restricted to B-cells and pDCs, its role in B-cell physiology remains to be clarified in the absence of recognition of any specific ligand. Human TLR10 is strongly related to TLR1 and 6 and associates with TLR2. While TLR10/TLR2 complexes recruit MyD88 after TLR2 ligand-induced stimulation, they fail to activate typical TLR-induced signalling, including NF-κB [105]. However, it seems likely that TLR10 is functional because TLR10 gene variants have been associated with susceptibility to asthma [105].

Thus, TLR expression is constitutive in human B-cells, assigning these cells a functional role in sensing infectious danger and participating in innate immunity; this function is well understood in mucosal surfaces but less so in other compartments. There are indications that B-cells display various arrays of TLR molecules that seem to characterize subsets. A question that is beginning to be addressed is whether TLR binding of pathogen-derived material tethers pathogen-derived Ags when the BcR is of low affinity (IgM). This may be the case for MZ B-cells, although this has not yet been ascertained because of the puzzling observation that MZ B-cells are CD21^hi^. The consequences of dual B-cell stimulation through a PRR (e.g., a TLR) and BcR deserve further exploration, particularly on Ab responses initiated by a TI Ag.

### Cross-talk between CD1d^+^ MZ-B-cells and NKT cells

**Figure 4:**
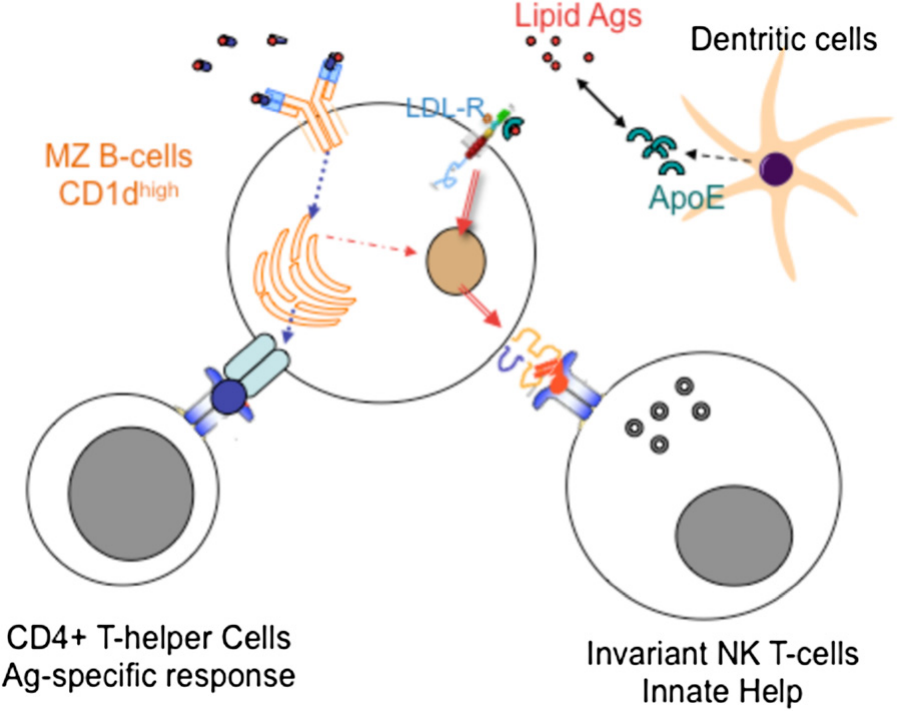
**MZ B-cells at the crossroad between BcR-dependent and CD1d-dependent B-cell responses to lipid antigens.** Through the expression of LDL-R, MZ B-cells capture and internalize aliprotein E (ApoE)-bound lipid Ags. Dendritic cells and macrophages in tissues secrete ApoE, which is present at low levels in human serum. ApoE-lipid Ag complexes are directed into the endosomal-lysosomal pathway and charged onto CD1d molecules. Exogenous lipids presented by CD1d interact with the invariant TCR of iNKT. These cognate interactions activate iNKT, which produces cytokines, and provide “innate help” to MZ B-cells. Because this internalisation pathway is independent on BcR, it might enhance humoral responses or induce pathogenic Abs [120]. The LDL-R-CD1d-dependent pathway for lipid Ag uptake by B-cells nevertheless provides a mechanism for the adjuvant effects of αGalCer [116]. Other studies suggest that αGalCer is routed to the endosomal-lysosomal pathway and charged onto CD1d molecules after BcR-mediated uptake of protein Ags linked to αGalCer [117], while BcR-mediated stimulation of human B-cells rapidly down-modulates CD1d expression [118].

Recent studies have demonstrated that in addition to the BcR-mediated uptake described for specific lipid Ags [116,117], B-cells can use an apolipoprotein-mediated pathway of lipid Ag uptake for presentation to innate-like NKT cells. Indeed, invariant NKT-cells (iNKT), a subset of NKT-cells, recognize exogenous and self-lipids as well as glycolipid Ags (LPS-free) presented by CD1d, a non-classical class I molecule. Through their characteristically high expression of CD1d, MZ B-cells may establish cognate interactions with iNKT in the MZ [118] and elicit a stronger lipid-driven NKT-cell stimulation than follicular B-cells [119]. The uptake of lipid Ags requires the expression of the low-density lipoprotein receptor (LDL-R) by B-cells, a BcR-independent uptake pathway [120] that probably favours the production of “polyreactive” (innate), and even self-reactive, Abs. While the BcR-mediated route facilitates the uptake of particulate lipid Ags and their transport to CD1d-containing endocytic vesicles, this pathway likely favours a more lipid-specific Ab response [118] (Figure 4). Although direct stimulation of TLR2, 7 and 9 by their ligands elicits IL-6, IL-10 and IFNβ (TLR9 only) production by MZ B-cells in mice, it does not result in cognate interactions with iNKT [121]. These results show that MZ B-cells may differentially contribute to humoral immunity according to their activation route and the nature of the Ag. Moreover, part of the innate Ab response depends on rapid but transient cognate interactions between MZ B-cells and NKT-cells. Recent studies have shown that impaired CD1d recycling in patients with systemic lupus erythematous, as compared with healthy individuals, causes defective B-cell mediated iNKT stimulation. This work highlights the physiological importance of B-cells in lipid presentation to iNKT during the innate immune response [122].

### The case of regulatory B-cells in mice and their potential equivalents in humans

B-cells are generally considered positive regulators of the immune response (their major regulator in Ig secretion function being Ig binding through the FcγRIIb itself). However, several regulatory B-cell subsets can negatively regulate these immune responses [123-125]. A general feature of these regulatory B-cells is to preferentially produce IL-10 upon appropriate stimulation [126-128], but this is not a unique feature. In particular, B10, a potent regulatory B-cell subset within the rare CD1d^hi^CD5^+^ B-cell subset of the spleen, has been shown to regulate acute inflammation and autoimmunity through the production of IL-10 [126,127]. Based on the expression of TIM-1 (*T-cell Ig domain and Mucin domain protein 1),* mouse B10 cells can be further distinguished from B-1a and MZ B-cells [129]. BAFF was recently shown to induce or expand B10 cells in mice [130].

Data obtained following B-cell depletion by treatment with Rituximab (a chimeric CD20 mAb), Ofatumumab or Ocrelizumab (two fully humanised anti-CD20 mAbs) in patients with autoimmune diseases or having undergone BM transplantation strongly support the existence of regulatory B-cells in humans [131]. However, it is not clear whether they constitute a distinct lineage or a subset, and have sufficient plasticity to change their effector functions into regulatory ones. IL-10-producing regulatory B-cells have been described in healthy individuals, as well as in patients with autoimmune disease [132,133]. In contrast to mice where B10 cells are naïve B-cells, human IL-10-producing B-cells have been characterised either as memory (CD27^+^) or immature transitional (CD38^hi^CD24^+^) B-cells. Only a population of human CD19^+^CD24^hi^CD38^hi^ B-cells was shown to exert regulatory functions on CD4^+^ T-cells [134]. Recent data from Iwata *et al.* partially reconcile these data by showing that both human B10 cells and their precursors belong to a CD24^hi^CD27^+^ B-cell population [133]. Thus, human B10 cells, similar to MZ B-cells, differ from mouse B10 cells by expressing a memory phenotype. The distinct organisation of MZ in mouse and human probably facilitates different interactions between MZM, DC and B-cell subsets [135]. In this context, it will be important to better identify BAFF-producing cells in human MZ because BAFF is likely involved in the differentiation or survival of human B10. Indeed, treatment of patients with multiple sclerosis (MS) with Atacicept (TACI-Ig), an antagonist of BAFF and APRIL, led to an unexpected increase in inflammatory activity and condemned the Atacicept trials in MS [136]. These data strongly support the notion that human B10 cells are also dependent on BAFF. While the various human B10 subsets produce IL-10 only after appropriate stimulation, a distinct population of CD20^+^CD27^+^CD43^+^CD11b^+^ B1 cells produce it spontaneously [137]. This population also down regulates the T-cell response. The respective role of these various IL-10-producing cells deserves to be extensively examined.

### CD21^lo/-^ B-cell populations: from aging to HIV infection

As previously discussed, ageing is accompanied by compromised immune responses and an increased propensity for autoimmunity. Besides reduced numbers of both hematopoietic stem cells entering into the lymphoid lineages and of MZ B-cells, accumulation of an exhausted B-cell population has recently been reported in aged mice [138]. With no surface CD21, CD23 and CD43, these CD19^hi^ SIgM^+^ BAFF-R^+^ B-cells differ from MZ, B1 and follicular B-cells. These B-cells further exhibit impaired functional responses to CD40 and BcR ligands, and to BAFF, and produce IgM, IL-4 and IL-10 only in response to TLR9 and TLR7 stimulation. Moreover, these B-cells are prone to induce IL-17 production by activated T-cells at the expense of T follicular helper cell generation, thus thwarting the interactions necessary for affinity maturation and memory cell formation [139].

In aged female mice and in mice presenting with autoimmune pathologies, an accumulation of CD21^lo/-^CD11c^+^ B-cells has also been described [140]. These CD11c^+^CD21^-^ B-cells, which are also CD5^+^ and CD138^+^, express high levels of CD95, CD80 and CD86. Unresponsive to BcR triggering, these B-cells secrete high levels of anti-chromatin IgG following TLR7 stimulation. Intact TLR7 signalling is required for the development of this population in mice. In humans, this population of CD21^-^ CD11c^+^ B-cells is CD5^hi^CD80^hi^CD86^hi^CD20^hi^CD23^-^, but CD27^hi^, and does not express any surface immunoglobulin; it therefore resembles plasmablast precursors [140]. A high frequency of circulating CD21^lo^ B-cells is associated with a propensity to autoimmunity, and the CD21^lo^CD11c^+^ population is found more frequently in the blood of elderly female patients with autoimmune disorders than in healthy age-matched individuals [11,141]. First described by Ehrhardt *et al.* as a unique population of memory B-cells expressing mutated BcR but not CD27, the FcRL4^+^ (*Fc receptor-like protein 4*) population normally resides in epithelial tissue-associated niches [142-145], but is expanded in the blood of patients with common variable immunodeficiency [146]. Because FcRL4 disrupts immune synapse formation and blocks antigen-induced BcR signalling, FcRL4^+^ B-cells are unresponsive to BcR ligands, but still responsive to CD40L, TLR9 agonists, IL 2 and IL 10 [147]. In viremic HIV-infected patients and patients chronically exposed to *plasmodium falciparum,* this population of FcRL4^+^CD27^-^ memory B-cells was found in the blood, where it progressively replaces conventional memory B-cells. In these HIV+ patients, FcRL4^+^ B-cells showed reduced proliferation and differentiation into plasma cells in response to BcR ligands, and also to cytokines and CD40L [148]. In light of this pathogen-induced loss of functions, Moir *et al*. called these cells “exhausted” memory B-cells. The role of FcRL4 in exhaustion is obvious: downregulation of FcRL4 expression by RNA interference in CD27^–^ memory B-cells in HIV-infected patients partially restores their capacity to respond to BcR stimulation and to produce HIV-specific antibodies [149]. At least in HIV-infected patients, these exhausted memory B-cells express high levels of PD-1 (*Programmed Death 1*) [148]. During pathogenic SIV infection in macaques, PD-1 drives the rapid and sustained loss of activated memory B-cells (CD21^lo^CD27^+^) in the so-called “rapid progressors”. This depletion, which constitutes an early predictor of disease progression, can be reversed by blockade of PD-1. In vivo blockade of PD1 in SIV-infected macaques have enhanced polyclonal and virus-specific Ab responses [150]. Whether similar mechanisms/pathways are responsible for CD21 downregulation in HIV-infected patients, patients with autoimmune diseases and aged individuals remains to be firmly established.

### Concluding remarks

In conclusion, B lymphocytes display hallmarks of three distinct functional categories of immune cells (Table 1): i) they bear a unique B-cell receptor for antigen on their membranes, which characterizes conventional B-cells and adaptive immunity; ii) they express CD40 on their surfaces, a property which is shared by most APC (though this is not a unique feature); iii) they express PAMP-ligands i.e. PRRs, which is considered a landmark of innate immune cells. Indeed, human B-cell subsets express distinct PRRs, including FcRs, complement receptors and TLRs. B-cells, despite being from a unique origin, have diversified into distinct subsets, the survival and functions of which are associated with their microanatomical locations. Accordingly, one can distinguish four main orientations. The first orientation comprises conventional, or follicular, B-cells that populate B-cell follicles in secondary lymphoid organs and mucosa, and correspond to the B2 lymphocytes in the mouse system. They principally use their capacity to bind amino acid-based Ags (although independent of APC and HLA in the conformational presentation) through the BcR and are specialised in the response to TD Ag. Qualified as naïve follicular B-cells before they encounter Ag, they evolve into activated B-cells that populate GCs and into memory or long-lived plasma cells that colonize specific areas around follicles and bone marrow, respectively. The second orientation concerns MZ non-conventional B-cells and B1-like B-cells, that can bind either peptide Ags through their BcR and/or non-peptide Ags such as lipids and polyosides or nucleic acids via a plethora of surface receptors. This subset is specialised for TI-Ab responses and preferentially home in spleen MZ. In contrast to follicular B-cells, which are highly recirculating cells, MZ B-cells have a restricted trafficking pattern. The third orientation is forwards regulatory B-cells (Breg), most of which produce high amounts of IL-10 upon appropriate stimulation. They have been identified both in mouse and humans, and parallel regulatory T-cells. Like MZ B-cells, Breg mainly locate in the spleen MZ where their survival and expansion are dependent on BAFF; In the fourth orientation, there is a novel complex of CD21^lo^ B-cell subsets that include at least three different populations: activated memory B-cells (CD21^lo^CD27^+^), plasmablast precursors (CD21^lo^CD27^hi^CD11c^+^CD138^+^) and “exhausted” memory B-cells (CD21^lo^CD27^lo^PD1^+^FcRL4^+^). Increased numbers of these B-cell subsets in blood have been preferentially reported in aged mice and humans, and in patients with autoimmune diseases or during chronic viral infection. B-cells belonging to these subsets harbour a non-functional BcR, and are frequently unresponsive to CD40L but strongly sensitive to TLR7 or TLR9 agonists. In healthy individuals, the latter CD21^lo^ population would preferentially reside in mucosa-associated tissues, immediately beneath the epithelium, likely sampling invasive pathogens. The identification of these new B-cell subsets, whose functions and survival are highly dependent on signals outside of those targeted at BcR, particularly homing, highlights the previously unexpected complexity of the B-cell compartment. It is thus a novel challenge in immunology to decipher the respective influence of innate and adaptive signals on the outcome of the B-cell responses in healthy individuals, and also during aging, autoimmune diseases, and chronic viral infection. The increasingly complex roles of B-cells are not yet fully characterised, nor are the differences between murine models and the human system fully determined. Further characterisation of the remaining mysteries of B-cell compartmentalisation is of key importance to human pathology, both in the fields of autoimmune disorders and onco-haematology.

**Table 1:** Summary of main phenotypic and functional characteristics of B-cell subsets

**Phenotypic marker**	**Functions and other characteristics**	**References**
**Marginal Zone B-cells**		
SIgM^hi^ SIgD^lo^CD27+ CD21^hi^	Mutated BcR in humans on >80% MZ B-cells	67-69,72
CD23-	TI-Ab response. Produced Low affinity IgM	
	CD21 modulates BCR signalling	141
CXCR5+ S1P1^hi^ S1P3^hi^	Preferential sequestration in MZ, shutling into follicles upon stimulation	55, 56, 58, 59
	BcR-independent transport of high MW Ags and virus particles into GC	56,83
	Present in periphery (blood, Lymph node, spleen) in humans	69
TLR 2> 1, 6; TLR10	Surface TLRs associated into functional TLR2/1 or TLR2/6 complexes	108, 109
TLR9>7	Endosomal TLR detecting unmethylated CpG DNA and ssRNA	
CD1c^hi^	Expression ↘ upon CD40L stimulation but ↗ after BcR triggering	115
LDL-R+	Binds and internalizes lipid Ags associated with Apo-E	117
CD1d^hi^	Presents lipid Ags to iNKT	116, 119-122
	CD1d expression decreases rapidly after BCR or CD40 activation	118
	Cognate interactions with iNKT, which in turn produce IL17 and IL22	119-122
BAFF-R++, TACI+	Strong expression of their ligand, BAFF, in MZ. Survival of MZ B-cells.	75
	Role of BAFF in the transient relocalisation of MZ B-cells into follicles	
**Follicular Naive B-cells**		
SIgM+ SIgD^hi^CD27- CD21+CD23^hi^	Unmutated BcR.	
CXCR5++ S1P1+ S1P3-	Preferential homing to follicles	56
TLR 2> 1, 6, TLR10	Surface TLRs associated into functional TLR2/1 or TLR2/6 complexes	108,109
TLR9>7	Endosomal TLR detecting unmethylated CpG DNA and ssRNA,	
CD1c+, CD1d^hi^	Functional role?	118
BAFF-R++, TACI+	Naive B-cell survival	
**Conventional Memory B-cells**		
SIgD-, SIgG/A>SIgM, CD27+	High affinity hypermutated BcR	
CD21^hi^CD23-	Role of CD21 in memory B-cell survival	141
TLR9++, TLR7+	↗ expression of TLR 7, 9 in memory B-cells compared to naive B-cells	7,113
BAFF-R+TACI+	BAFF preferentially enhances memory B-cell chemotaxis to CXCL13	86
**Regulatory B-cells**		
CD1d^hi^ CD5+ TIM-1+	B10:IL10-producing B-cells in mice. Located in/near MZ	123,124
	Expanded by BAFF in mice	130
CD27+	IL10-producing memory B-cells in humans	127, 132-134
CD19+CD24^hi^ CD38^hi^	IL10-producing transitional B-cells in humans. Regulatory functions on CD4+ T-cells	134
**CD21**^**lo/-**^**B-cells**		
CD19+ SIgM+ BAFF-R+ CD21-	No response to BcR, CD40 or BAFF-R stimulation	134
CD23-CD43-	Production of IgM, IL10 and IL4 in response to TLR9, 7 stimulation	
	Induce IL17 production by activated T-cells	115
CD21^lo^ CD11c+ CD5+ CD138+	Present in aged and autoimmune mice. Pre-plasmablasts?	157
CD95^hi^ CD80^hi^ CD86^hi^	No response to BcR stimulation but produced IgG after TLR7 triggering	
CD20^hi^ CD21^lo^ CD11c+ CD5^hi^CD27^hi^	Present in blood of elderly female autoimmune patients	132,133
CD23-SIg-CD80^hi^CD86^hi^	Pre-plasmablasts?	
CD21^lo^CD27+	Activated B-cells or pre-plasmablasts present in HIV-infected patients	148-150
CD21^lo^CD27-PD1+ FcRL4+	Exhausted tissue-like memory B-cells present in HIV-infected patients	148-150

## Competing interests

The authors declare no competing of interest with respect of this study; neither do they have financial and commercial interests with the industry. Further, they have no conflict of interest of any type with any issue related with their 5 year past and present research.

## Authors’ contributions

OG and YR co-wrote the manuscript; FC and BP co-edited the manuscript; GB, GB, FC and SD contributed original data that inspired the manuscript, and drafted the illustrations. All authors read and approved the final manuscript.
